# Akt+ IKKα/β+ Rab5+ Signalosome Mediate the Endosomal Recruitment of Sec61 and Contribute to Cross-Presentation in Bone Marrow Precursor Cells

**DOI:** 10.3390/vaccines8030539

**Published:** 2020-09-17

**Authors:** Dan Dan Xu, Chun Fang Hu, Xiang You, Nan Nan Lu, Feng Guang Gao

**Affiliations:** Department of Basic Medical Sciences, School of Medicine, Xiamen University, Xiamen 361102, China; 18750226531@163.com (D.D.X.); huchunfangxf@163.com (C.F.H.); yx_you@xmu.edu.cn (X.Y.); 18232150268@163.com (N.N.L.)

**Keywords:** Akt, Sec61, bone marrow precursor cells, cross-presentation, IKKα, IKKβ

## Abstract

Cross-presentation in dendritic cells (DC) requires the endosomal relocations of internalized antigens and the endoplasmic reticulum protein Sec61. Despite the fact that endotoxin-containing pathogen and endotoxin-free antigen have different effects on protein kinase B (Akt) and I-kappa B Kinase α/β (IKKα/β) activation, the exact roles of Akt phosphorylation, IKKα or IKKβ activation in endotoxin-containing pathogen-derived cross-presentation are poorly understood. In this study, endotoxin-free ovalbumin supplemented with endotoxin was used as a model pathogen. We investigated the effects of endotoxin-containing pathogen and endotoxin-free antigen on Akt phosphorylation, IKKα/β activation, and explored the mechanisms that the endotoxin-containing pathogen orchestrating the endosomal recruitment of Sec61 of the cross-presentation in bone marrow precursor cells (BMPC). We demonstrated that endotoxin-containing pathogen and endotoxin-free antigen efficiently induced the phosphorylation of Akt-IKKα/β and Akt-IKKα, respectively. Endotoxin-containing pathogen derived Akt+ IKKα/β+ Rab5+ signalosome, together with augmented the recruitment of Sec61 toward endosome, lead to the increased cross-presentation in BMPC. Importantly, the endosomal recruitment of Sec61 was partly mediated by the formation of Akt+ IKKα/β+ signalosome. Thus, these data suggest that Akt+ IKKα/β+ Rab5+ signalosome contribute to endotoxin-containing pathogen-induced the endosomal recruitment of Sec61 and the superior efficacy of cross-presentation in BMPC.

## 1. Introduction

As dendritic cells (DC) differentiated from bone marrow precursor cells (BMPC) with lacking or inappropriate encounter with antigen might result in the absence or the silencing of an immune response, studies based on ex vivo generated autologous DC under controlled condition are urgently needed [1]. Surface molecules such as mannose receptor (MR), lectin, CD40, langerin, heat shock protein mediated cross-presentation allows DC presenting extracellular antigen and inducing protective immunity against intracellular microbes infection and tumors [1,2]. Once DC recruit uptaked extracellular antigen toward endosome, cross-presentation occur via vacuolar or endosome-to-cytosol pathway [3,4,5,6]. During the process of cross-presentation, the antigen within endosome is degraded by lysosomal proteases or degraded in the cytosol by cytosolic proteinase [3,4,7,8]. All these findings indicate that antigen resident endosome is a main compartment for DC cross-presentation.

Protein kinase B (Akt), a serine/threonine kinase, anchor to the plasma membrane by interacting with membrane lipid products such as phosphatidylinositol 4,5-diphosphate (PIP2) and phosphatidylinositol 3,4,5-triphosphate (PIP3) [9]. The blocking of Akt activation perturbs endocytic uptaking and inhibits DC cross-presentation [10]. Whereas lectin binding to immunoglobulin (Ig) variable region provides the activating signals for extracellular signal-regulated kinase 1/2 (Erk1/2), Akt, and phosphoinositide-specific phospholipase C γ2 (PLCγ2) [11]. Akt exerts its effects by phosphorylating a variety of substrates, which include Bcl-2 agonist of cell death (BAD), cysteinyl aspartate specific proteinase 9 (Caspase 9), endothelial nitric-oxide synthase (eNOS), I-kappa B Kinase (IKK), and transcription factors of the nuclear factor κB (NF-κB) [12,13,14]. Toll like receptor 4 (TLR4)-NF-κB signaling increase the endosomal translocation of MR-internalized antigens and lead to augmented cross-presentation in DC [5,15]. As the complement membrane attack complexes activate noncanonical NF-κB by forming an Akt+ NF-κB-inducing kinase (NIK)+ signalosome on Rab5+ endosome [12], the exact effects of endotoxin-containing pathogen on Akt activation and the roles of Akt+ IKKα/β+ signalosome in DC cross-presentation are poorly understood.

In the endosome-to-cytosol pathway, antigens in the endosome need to be exported into the cytosol [7,8]. During this process, valosin-containing protein p97 (p97) relocate toward endosome to provide the driving force for the transport of misfolded proteins [4,5,16,17]. Meanwhile, Sec61 was documented to relocate from the endoplasmic reticulum (ER) toward the antigen resident phagosome to provide the driving force [18,19]. However, up to now, little is known about the effects of Akt phosphorylation and IKK activation on the endosomal recruitment of Sec61 in endotoxin-containing pathogen-derived cross-presentation in DC.

In the present study, endotoxin-free ovalbumin (OVA) supplemented with endotoxin was used as endotoxin-containing pathogen. We investigated the effects of endotoxin-containing pathogen and endotoxin-free antigen on Akt phosphorylation and IKKα/β activation, and explored the mechanism that endotoxin-containing pathogen orchestrating the endosomal recruitment of Sec61 in the process of the cross-presentation in BMPC. We demonstrated that the treatments with endotoxin-containing pathogen and endotoxin-free antigen induced the phosphorylations of Akt-IKKα/β or Akt-IKKα, respectively. Interestingly, Akt+ IKKα/β+ signalosome obviously mediated the relocation of Sec61 toward the endosome. Thus, these findings suggest that Akt+ IKKα/β+ Rab5+ signalosome contribute to model pathogen induced the endosomal recruitment of Sec61 and the superior efficacy of endotoxin-containing pathogen-derived the cross-presentation in BMPC.

## 2. Materials and Methods

### 2.1. Mice

We bought specific pathogen-free C57BL/6 mice (female, 4–6 weeks old) from the Shanghai Laboratory Animal Center. The mice were kept at the Xiamen University Laboratory Animal Center. Animals were housed with an inverse 12 h day–night cycle with lights on at 8:30 p.m. in a temperature and humidity controlled room. All cages contained wood shavings, bedding, and a cardboard tube for environmental enrichment. The Ethics number is “XMULAC20170016”, which was approved by the Ethics Committee of the Xiamen University.

### 2.2. Reagents and Antibodies

Reagents used in this study were from the following companies: Sigma-Aldrich (St. Louis, MO, USA) for dimethyl sulfoxide (DMSO) and endotoxin from *Escherichia coli*. Hyglos GmbH (Regensburg, Germany) for endotoxin-free EndoGrade-ovalbumin. PeproTech (Rocky Hill, NJ, USA) for murine granulocyte-macrophage colony-stimulating factor (GM-CSF) and interleukin-4 (IL-4). Cayman Chemical (Ann Arbor, MI, USA) for Bay11-7082, wortmannin. Phycoerythrin (PE)-conjugated 25-D1.16, (#141604) was from BioLegend (San Diego, CA, USA). Cell Signaling Technology (Beverly, MA, USA) for antibodies to IKKα (D3W6N, #61294; #2682), IKKβ (D30C6, #8943; L570, #2678), Akt (11E7, #4685; 40D4, #2920), phosphorylated Akt (Ser473) (#9271), phosphorylated IKKα/β (Ser176/180) (16A6, #2697), horseradish peroxidase (HRP) conjugate secondary antibody (#7074). Abcam (Cambridge, UK) for Rab5 (#ab218624, #ab18211). Santa Cruz Biotechnology (Dallas, TX, USA) for siRNAs of Akt (sc-43610), IKKα (sc-29366), IKKβ (sc-35645), and control siRNA (sc-37007), antibodies of Rab5 (D-11, sc-46692), Sec61α (G-20, sc-12322), glyceraldehyde-3-phosphate dehydrogenase (GAPDH) (6C5, sc-32233), and Protein A/G Plus-Agarose, Transfect reagent. HyClone (Logan, UT, USA) for BMPC medium and fetal bovine serum (FBS).

### 2.3. Murine Bone Marrow Precursor Cell Culture

BMPC was induced according to previous description [20]. Briefly, C57BL/6 mice were sacrificed and the intact bone was dissected. Repeated flushing with complete RPMI-1640 media (HyClone) was performed to harvest the bone marrow. Then, red blood cells were depleted from mononuclear cells of the bone marrow. Mononuclear cells were then cultured in 3.5 cm dishes (Thermo Scientific) for further 4 days at a density of 1 × 10^6^ cells/mL with GM-CSF and IL-4 at the final concentrations of 10 ng/mL and 1 ng/mL, respectively. After gentle wash to remove non-adherent cells, the remaining adherent cluster was referred to BMPC.

### 2.4. RNAi Transfection

RNAi transfection was performed according to the guideline of the manufacturer. Briefly, 2–8 µL of siRNA duplex (20–80 pmols siRNA) or Transfection Reagent (sc-29528) were diluted into 100 µL siRNA Transfection Medium (sc-36868) and referred as buffer A and B respectively. Then, buffer A and B was gently mixed and incubated for 45 min at room temperature and referred as buffer C. BMPC was washed with Transfection Medium (sc-36868), overlaid siRNA Transfection Medium containing buffer C and incubated for further 7 h. After incubation, complete medium containing 20% FBS was appended and the BMPC was further cultured for 18–24 h. Then, the cells were cultured with fresh medium for 48–72 h. The effect of indicative siRNA in BMPC was validated in supplementary data.

### 2.5. Bone Marrow Precursor Cell Treatments

Endotoxin-free ovalbumin (EndoGrade-ovalbumin) supplemented with 1 ng/mL lipopolysaccharide (LPS) at the final concentration was used as endotoxin-containing model pathogen. To investigate the effects of endotoxin-containing pathogen on Akt/IKKα/β activation and the endosomal translocation of Sec61, BMPC was incubated with endotoxin-containing model pathogen or endotoxin-free ovalbumin at the final concentration of 50 μg/mL. To inhibit related kinase activities, BMPC was pretreated with wortmannin (5 μM), Bay11-7082 (5 μM) or siRNA transfection prior to the incubation with endotoxin-containing model pathogen (50 μg/mL). For cytometric analyses of cross-presented OVA, the BMPC was conferred with endotoxin-containing model pathogen for 7 h.

### 2.6. Flow Cytometric Measurements

The effect of kinase inhibition on the cross-presentation of BMPC was determined via flow cytometry [20]. Briefly, BMPC was firstly incubated with 10% BSA and further blocked with CD16/32 antibody. Then, the cells were stained with antibody for OVA-derived peptide SIINFEKL bound to H-2Kb of major histocompatibility complex (MHC) class Ⅰ, at a final concentration of ≤0.125 µg per million cells. After a thorough wash with PBS, unbound antibody was removed. Flow cytometry was done at the wavelength 488 nm with FACSCalibur and data were analyzed with CellQuest software. The reagent was titrated for optimal performance for each application.

### 2.7. Co-Immunoprecipitation

Co-Immunoprecipitation (Co-IP) was performed according to previous description [21]. Briefly, BMPC conferred with above treatments was harvested and lysed in the buffer of Pierce™ Co-Immunoprecipitation Kit (Cat. 26149). The lysates were then incubated with 20 μL/mL Protein A/G agarose beads for 1 h for pre-clear. Then, indicated primary antibody or control IgG was incubated with the supernatant in RIPA buffer overnight at 4 °C, and further followed by the addition of 20 µL/mL Protein A/G agarose beads. Thorough wash was performed with RIPA buffer and co-immunoprecipitates were re-suspended in sodium dodecyl sulfate (SDS) sample buffer, boiled for 5 min. Proteins were electrophoretically transferred to polyvinylidene fluoride (PVDF) membranes and subjected to Western blot analysis using the indicated antibodies.

### 2.8. Western Blots

Whole cellular lysates or the output of Co-IP was subjected to 7–10% SDS polyacrylamide gel electrophoresis (SDS-PAGE) according to previous description [21]. Briefly, proteins were electrophoretically transferred onto a PVDF membrane (Millipore). Then, the membrane was blocked with 5% evaporated milk in Tris base SDS–0.05% Tween and further incubated with primary antibody. After a thorough wash, the bounded primary antibody was detected by peroxidase-conjugated secondary antibody. In the end, the bound secondary antibody was revealed by ECL western blot reagents (Advansta, Menlo Park, CA, USA) according to the manufacturer’s directions. The loading control was GAPDH.

### 2.9. Statistical Analysis

Cross-presented OVA was determined by flow cytometric analyses and presented as positive percentage and mean of fluorescence index (MFI) of analyzed cell population. Data are presented as the mean ± SEM. The Gaussian distribution of the data was evaluated by Shapiro–Wilk normality test. Then, statistical significance was assessed by Student’s *t*-test, with a value of *p* < 0.05 considered statistically significant. 

## 3. Results

### 3.1. Endotoxin-Containing Pathogen and Endotoxin-Free Antigen Induce Different Effect on the Phosphorylation of Akt, IKKα/β in Bone Marrow Precursor Cells

**Figure 1:**
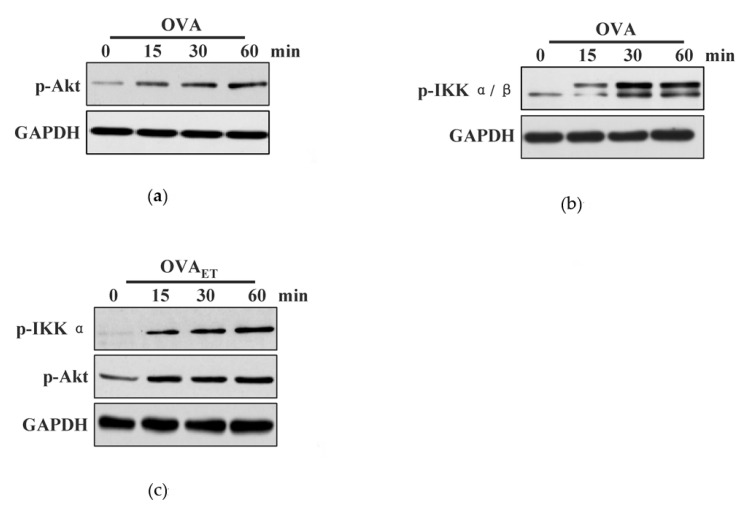
Endotoxin-containing pathogen and endotoxin-free antigen induce different effects on the phosphorylation of protein kinase B (Akt) and I-kappa B Kinase α (IKKα) in bone marrow precursor cells. (**a**–**c**) Murine granulocyte-macrophage colony-stimulating factor (GM-CSF) and interleukin-4 (IL-4) treated bone marrow precursor cells (BMPC) was incubated with endotoxin-containing pathogen ovalbumin (**a**,**b**) or endotoxin-free ovalbumin (**c**) (50 μg/mL) for indicated periods. The effects of endotoxin-containing pathogen and endotoxin-free ovalbumin on the phosphorylation of Akt and IKKα/β were determined by western blot analyses. GAPDH was used as an internal control. One representative from 3 independent experiments is shown. OVA: endotoxin-containing pathogen ovalbumin; OVA_ET_: endotoxin-free ovalbumin.

Cross-presentation require antigen entering into endosome, a specific intracellular pathway that determined by the mechanism of antigen uptake [22]. In despite the fact that antigen-presenting cell prime antigen-specific naïve T cells via phosphoinositide 3-kinase (PI3K)-Akt/p38-NF-κB signaling [23], the exact roles of these activated kinases in the uptake of exogenous antigen are unclear. To address this issue, we incubated GM-CSF and IL-4 treated BMPC with endotoxin-containing pathogen OVA and monitored Akt and IKKα/β phosphorylation by western blot. Whereas the treatment with endotoxin-containing pathogen efficiently induced the phosphorylation of Akt (Figure 1a), IKKα/β assay revealed that the levels of phosphorylated IKKα/β increased from 15 to 60 min (Figure 1b). When BMPC was incubated with OVA, the increased co-localized spots of phosphorylated Akt and IKKα/β with Rab5 can also be observed by the immuno-fluorescent microscope (Figure S1).

Burgdorf et al. documented that efficient cross-presentation require endotoxin-induced, TLR4 dependent the relocation of the transporter associated with antigen processing (TAP) [6]. As Akt signaling is essential for TLR-induced rapid activation in DC [22], we further incubated BMPC with endotoxin-containing pathogen and endotoxin-free OVA to clarify the exact roles of pathogen-containing endotoxin and endotoxin-free antigen in the process of cross-presentation in BMPC. Importantly, endotoxin-free OVA itself induced Akt phosphorylation in lacking endotoxin’s condition (Figure 1c). Interestingly, phosphorylated IKKα, but not phosphorylated IKKβ can only be monitored in endotoxin-free antigen-loaded condition (Figure 1c). As Akt activation is involved in complement membrane attack complexes inducing the IKKα phosphorylation [12], the effects that endotoxin-free antigen on the phosphorylation of IKKα and Akt indicate that the activation of IKKα and Akt might play specific roles in endotoxin-containing pathogen-derived the cross-presentation in BMPC.

### 3.2. Akt Phosphorylation Is Essential for Endotoxin-Containing Pathogen-Derived Cross-Presentation in Bone Marrow Precursor Cells

**Figure 2:**
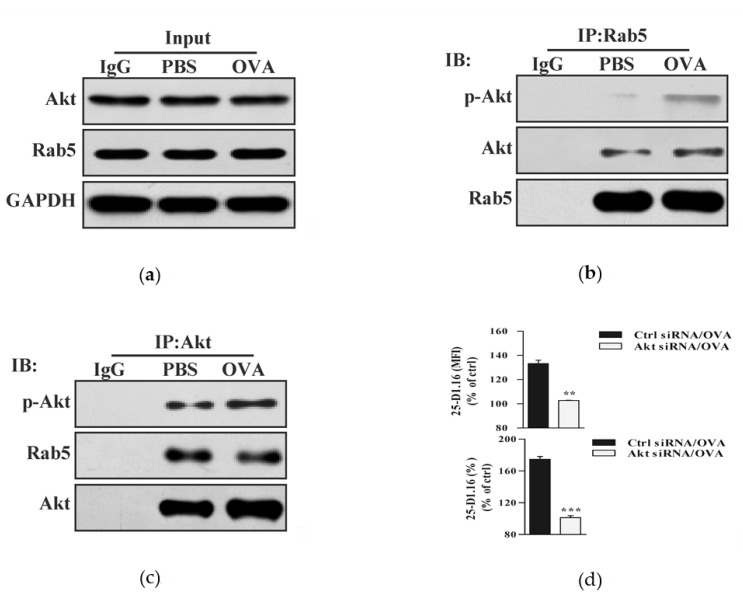
Akt phosphorylation is essential for endotoxin-containing pathogen-derived cross-presentation in bone marrow precursor cells. (**a**–**c**) Murine BMPC was incubated with endotoxin-containing pathogen ovalbumin (50 μg/mL). Akt expression (**a**) and interaction of phosphorylated Akt-Rab5 (**b**,**c**) were determined by co-immunoprecipitation (Co-IP) with Rab5 (**b**) or Akt (**c**) antibody. Isotype IgG was used as negative control. Whole cellular protein was used as input control. (**d**) Akt deficient and control BMPC was incubated with endotoxin-containing pathogen ovalbumin (50 μg/mL) and cross-presented OVA was assessed by flow cytometric analyses. Data are presented as the mean ± SEM, ** *p* < 0.01, *** *p* < 0.001, Student *t*-test. One representative from three independent experiments is shown. OVA: endotoxin-containing pathogen ovalbumin.

Whereas lysosome is responsible for presenting internalized antigen on MHC class Ⅱ molecules [22], endosome is the main compartment for cross-presenting excellular antigen on MHC Ⅰ molecules [6,15]. We demonstrated that the treatments with endotoxin-containing pathogen and endotoxin-free antigen induced Akt phosphorylation (Figure 1). As efficient cross-presentation requires endotoxin-induced signaling [6], we wondered whether Akt phosphorylation facilitates endotoxin-containing pathogen-derived the endosomal recruitment of phosphorylated Akt. To address this issue, we incubated BMPC with endotoxin-containing pathogen, and assessed the interaction of phosphorylated Akt with Rab5. Whereas endotoxin-containing pathogen had no effect on Akt and Rab5 expression (Figure 2a), an obvious interaction of phosphorylated Akt with Rab5 can be monitored in the output of Rab5 antibody-anticipated Co-IP (Figure 2b). Similarly, endotoxin-containing pathogen-increased the interaction of phosphorylated Akt and Rab5 can also be easily achieved in the output of Akt antibody-anticipated Co-IP (Figure 2c).

Early endosome is a subcellular compartment for cross-presented antigen target into [22,24,25]. Given that the silencing of Akt efficiently inhibited endotoxin-containing pathogen-derived Akt activation (Figure S2), we incubated Akt-deficient and control BMPC with endotoxin-containing pathogen and monitored cross-presented OVA by flow cytometric analyses. The data showed that the deficiency of Akt decreased endotoxin-containing pathogen-derived cross-presented OVA in BMPC (Figure 2d). When Akt activation was inhibited by the treatment with wortmannin, the decreased co-localized spots of cross-presented OVA with Rab5 was also observed by the immuno-fluorescent microscope (Figure S3a,c). All these findings indicate that Akt activation play a pivotal role in endotoxin-containing pathogen-derived cross-presentation in BMPC.

### 3.3. Both IKKα and IKKβ Phosphorylation Augment Endotoxin-Containing Pathogen-Derived Cross-Presentation in Bone Marrow Precursor Cells

**Figure 3:**
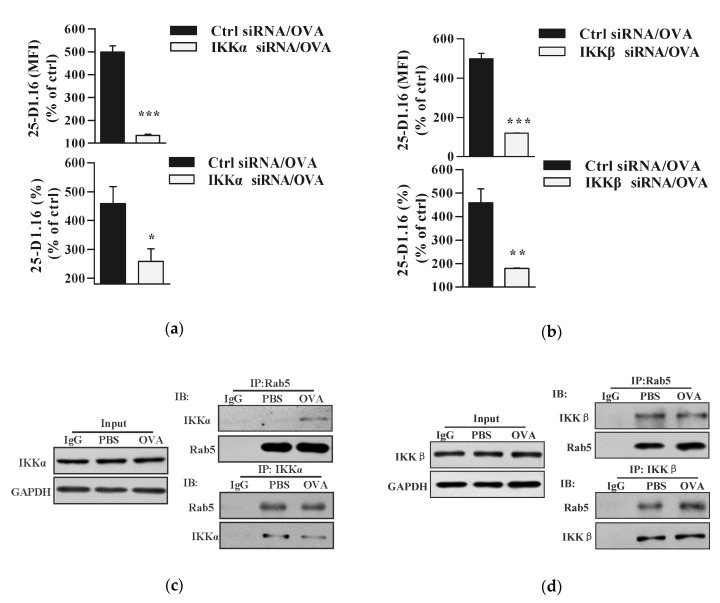
Both IKKα and IKKβ phosphorylation augment endotoxin-containing pathogen-derived cross-presentation in bone marrow precursor cells. (**a**,**b**) Murine IKKα/IKKβ deficient or control BMPC was incubated with endotoxin-containing pathogen ovalbumin (50 μg/mL) and cross-presented OVA was assessed by flow cytometric analyses. (**c**,**d**) Murine BMPC was incubated with endotoxin-containing pathogen ovalbumin (50 μg/mL) and the interaction of IKKα-Rab5 (**c**) or IKKβ-Rab5 (**d**) was investigated by Co-IP with Rab5, IKKα or IKKβ antibody, respectively. Isotype IgG was used as negative control. Whole cellular protein was used as input control. Data are presented as the mean ± SEM, * *p* < 0.05, ** *p* < 0.01, *** *p* < 0.001, Student *t*-test. One representative from three independent experiments is shown. OVA: endotoxin-containing pathogen ovalbumin.

TLR4 signaling, which results in IκB kinase degradation, play vital roles in the cross-presentation in DC [6,15,19,26]. As endotoxin-free antigen and endotoxin-containing pathogen had different effect on IKKα and IKKβ phosphorylation (Figure 1), we wondered whether IKKα and IKKβ are all needed in endotoxin-containing pathogen-derived cross-presentation. To the end, we incubated IKKα or IKKβ deficient BMPC with endotoxin-containing pathogen and assessed the cross-presented OVA by cytometric analyses. Given that the silencing of IKKα or IKKβ efficiently inhibited endotoxin-containing pathogen-induced its phosphorylation (Figure S2), endotoxin-containing pathogen-derived cross-presented OVA was obviously decreased by the deficiency of IKKα (Figure 3a). Subsequent IKKβ-deficient BMPC cytometric analyses revealed that endotoxin-containing pathogen-derived cross-presented OVA was also inhibited by the deficiency of IKKβ (Figure 3b). Meanwhile, the co-localized spots of cross-presented OVA with Rab5 were efficiently attenuated by Bay11-7082 pretreatment (Figure S3b,d). All these findings indicate that IKKα and IKKβ are pivotal molecules for endotoxin-containing pathogen-derived cross-presentation in BMPC.

Complement membrane attack complexes form an Akt+ NIK+ Rab5+ signalosome and activate non-canonical NF-κB [12]. Virus loading was also reported to induce IKKα/β phosphorylation [27]. To dissect the roles of IKKα and IKKβ in endotoxin-containing pathogen-derived cross-presentation, we incubated BMPC with endotoxin-containing pathogen and monitored the potential interaction of IKKα or IKKβ with antigen-containing endosome. In despite the fact that endotoxin-containing pathogen had no effect on IKKα expression, an obvious interaction of IKKα-Rab5 or Rab5-IKKα was monitored in model pathogen-loaded condition (Figure 3c). Meanwhile, the interaction of IKKβ-Rab5 and Rab5-IKKβ was also achieved in endotoxin-containing pathogen-loaded condition (Figure 3d). As Rab5 is a marker of early endosome, these findings indicate that endotoxin-containing pathogen promote the formation of IKKα+ Rab5+ or IKKβ+ Rab5+ signalosome. As the deficiencies of IKKα and IKKβ inhibited pathogen-derived cross-presentation (Figure 3a,b), the interaction of IKKα-Rab5 and IKKβ-Rab5 indicate that IKKα+ Rab5+ and IKKβ+ Rab5+ signalosome facilitate the process of cross-presentation in BMPC.

### 3.4. Endotoxin-Containing Pathogen Promotes the Formation of Akt+ Ikkα/Β+ Rab5+ Signalosome in Bone Marrow Precursor Cells

**Figure 4:**
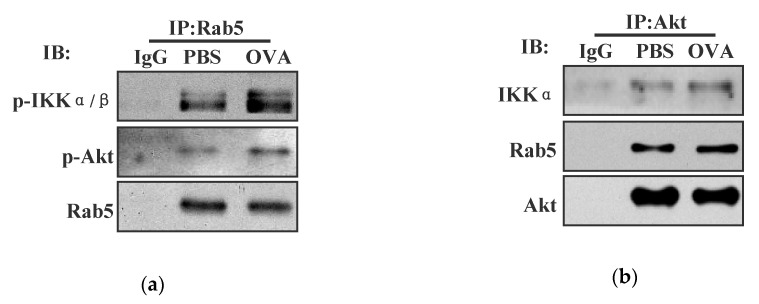

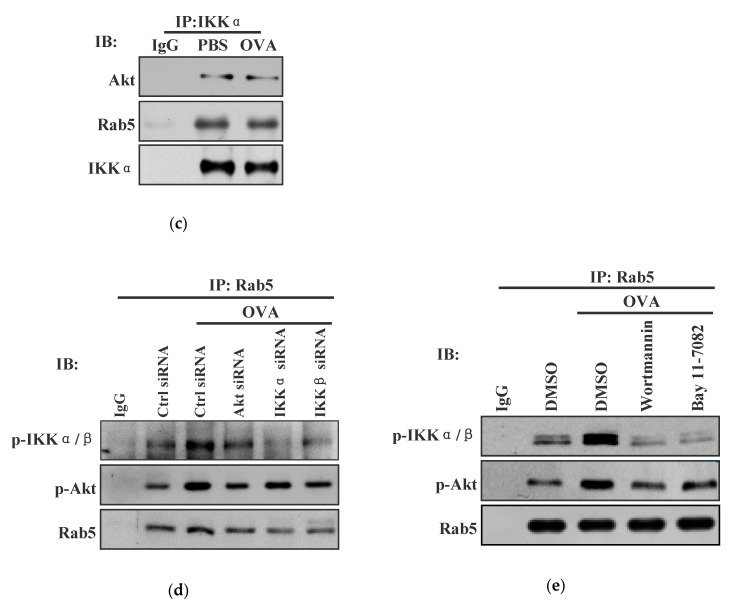
Endotoxin-containing pathogen promotes the formation of Akt+ IKKα/β+ Rab5+ signalosome in bone marrow precursor cells. (**a**–**c**) Murine BMPC was incubated with endotoxin-containing pathogen ovalbumin (50 μg/mL) and the interaction of Akt-IKKα/β-Rab5 was assessed by Co-IP with Rab5 (A), Akt (B), or IKKα (**c**) antibody. Isotype IgG was used as negative control. Murine scramble, Akt/IKKα/IKKβ deficient (**d**), or Bay11-7082/wortmannin (5 μmol/L) pretreated (**e**) BMPC was incubated with endotoxin-containing pathogen ovalbumin (50 μg/mL). The interaction of phosphorylated IKKα/β with Rab5, phosphorylated Akt with Rab5 were investigated by Co-IP with Rab5 antibody. Isotype IgG was used as negative control. Control siRNA or DMSO with ovalbumin was used as endotoxin-containing pathogen control. Control siRNA or DMSO without ovalbumin was used as scramble control. One representative from three independent experiments is shown. OVA: endotoxin-containing pathogen ovalbumin.

As endotoxin-containing pathogen and endotoxin-free antigen had different effects on IKKα/β activation and on the cross-presentation in BMPC (Figure 2 and Figure 3), we wondered whether the loading of endotoxin-containing pathogen could form Akt+ IKKα/β+ Rab5+ signalosome. To address this issue, we incubated BMPC with endotoxin-containing pathogen and assessed the interaction of Akt-IKKα/β with antigen-containing endosomes. In consistent with the finding that endotoxin-containing pathogen promoted Akt and IKKα/β phosphorylation (Figure 1), an obvious interaction of phosphorylated Akt with Rab5 and phosphorylated IKKα/β with Rab5 can be monitored in endotoxin-containing pathogen-loaded condition (Figure 4a). Importantly, the interaction of IKKα with Akt and Rab5 with Akt was also achieved in the output of Akt antibody-mediated Co-IP (Figure 4b). Similarly, the interaction of Rab5 with IKKα and Akt with IKKα was reconfirmed in IKKα-mediated Co-IP (Figure 4c). All these findings indicate that the loading of endotoxin-containing pathogen promote the formation of Akt+ IKKα/β+ Rab5+ signalosome in BMPC.

To investigate the role of Akt and IKKα/β activation in endotoxin-containing pathogen-promoted the formation of Akt+ IKKα/β+ Rab5+ signalosome, Akt/IKKα/β deficient or wortmannin/Bay11-7082 pretreated BMPC was incubated with endotoxin-containing pathogen and the interaction of Akt-IKKα/β-Rab5 was re-assessed by Co-IP analyses. In consistent with the finding that endotoxin-containing pathogen-augmented the formation of Akt+ IKKα/β+ Rab5+ signalosome (Figure 4a–c), an efficient interaction of phosphorylated Akt with Rab5 and phosphorylated IKKα/β with Rab5 can be monitored in endotoxin-containing pathogen-loaded condition (Figure 4d,e). This interaction was abolished by the deficiencies of Akt, IKKα, and IKKβ (Figure 4d). Moreover, pathogen-increased the interaction of phosphorylated Akt with Rab5 and phosphorylated IKKα/β with Rab5 were also alleviated by the pretreatments with wortmannin and Bay11-7082 (Figure 4e). The observation by Immuno-fluorescent microscope revealed that the co-localized spots of phosphorylated Akt-Rab5 or p-IKKα/β-Rab5 were exactly attenuated by the pretreatments with wortmannin and Bay11-7082 (Figure S4). Whereas the co-localized spots of phosphorylated Akt with Rab5 were decreased by Bay11-7082 pre-treatment, the co-localized spots of phosphorylated IKKα/β with Rab5 were also inhibited by the treatment with wortmannin (Figure S4). All these findings indicate that Akt and IKKα/β phosphorylation are important for endotoxin-containing pathogen-promoted the formation of Akt+ IKKα/β+ Rab5+ signalosome.

### 3.5. Akt+ IKKα/β+ Rab5+ Signalosome Facilitates Endotoxin-Containing Pathogen-Induced the Endosomal Relocation of Sec61α

**Figure 5:**
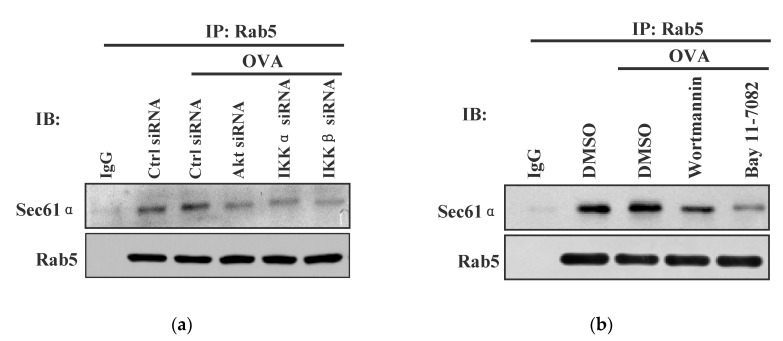
Akt+ IKKα/β+ Rab5+ signalosome facilitates endotoxin-containing pathogen-derived the relocation of Sec61α. Murine scramble, Akt/IKKα/IKKβ deficient (**a**), or Bay11-7082/wortmannin (5 μmol/L) pretreated (**b**) BMPC was incubated with model pathogen ovalbumin (50 μg/mL). The interaction of Sec61α with Rab5 was investigated by Co-IP with Rab5 antibody. Isotype IgG was used as negative control. Control siRNA or DMSO with ovalbumin was used as endotoxin-containing pathogen control. Control siRNA or DMSO without ovalbumin was used as scramble control. One representative from three independent experiments is shown. Rab5: early endosome marker; OVA: endotoxin-containing pathogen ovalbumin.

In the process of DC cross-presentation, p97 and TAP relocate to the endosome to transport the internalized antigen [4,17,18]. Sec61, another protein in the ER, relocate toward antigen-containing endosome and provide the energy for the event of transmembrane movement [18,19]. To investigate the potential role of Akt+ IKKα/β+ Rab5+ signalosome in endotoxin-containing pathogen-derived the endosomal relocation of Sec61, Akt/IKKα/β deficient or wortmannin/Bay11-7082 pretreated BMPC was incubated with endotoxin-containing pathogen and the interaction of Sec61α with Rab5 was re-assessed by Co-IP analyses. Whereas the loading of endotoxin-containing pathogen-increased the interaction of Sec61α-Rab5, the interaction of Sec61α with Rab5 was efficiently inhibited by the deficiencies of Akt, IKKα, and IKKβ (Figure 5a). Moreover, the interaction of Sec61α with Rab5 was also abrogated by the pretreatments with wortmannin and Bay11-7082 (Figure 5b). Endotoxin-containing pathogen-increased the interaction of Sec61β with Rab5 was also abrogated by the pretreatments with wortmannin and Bay11-7082 (Figure S5). The observation by immuno-fluorescent microscope revealed that the co-localized spots of Sec61β with Rab5 were exactly attenuated by the deficiencies of Akt, IKKα, and IKKβ (Figure S6). All these findings indicate that Akt+ IKKα/β+ Rab5+ signalosome play important roles in endotoxin-containing pathogen-derived the endosomal relocation of Sec61 in BMPC.

## 4. Discussion

In this study, endotoxin-free OVA supplemented with or without endotoxin was used as endotoxin-containing model pathogen or endotoxin-free antigen, respectively. We investigated the effects of endotoxin-containing pathogen and endotoxin-free antigen on Akt phosphorylation and IKKα/β activation. We further explored the mechanism that endotoxin-containing pathogen orchestrating the endosomal recruitment of Sec61 in the process of cross-presentation in BMPC. We demonstrated that endotoxin-containing pathogen or endotoxin-free antigen induced the phosphorylation of Akt/IKKα/β and phosphorylation of Akt/IKKα, respectively. Pathogen loading-derived Akt+ IKKα/β+ Rab5+ signalosome, together with augmented the recruitment of Sec61 toward endosome, lead to the increased cross-presentation in BMPC. All these findings indicate that the increased recruitment of Akt+ IKKα/β+ signalosome and Sec61 toward Ag-containing vesicles contribute to the superior efficacy of cross-presentation in endotoxin-containing pathogen-loaded BMPC.

Ubiquitination, an important event for protein relocation and degradation, is emerging as a new mechanism for immune regulation [28]. In the present study, in despite the fact that the loading of endotoxin-containing pathogen facilitate the formation of Akt+ IKKα/β+ Rab5+ signalosome, the exact mechanisms that the endosomal relocation of Akt and IKK promoting endotoxin-containing pathogen-derived cross-presentation are still to be clarified. X-linked inhibitor-of-apoptosis protein (XIAP), a physiological substrate of Akt, interact with phosphorylated Akt at serine 87 [29]. These effects reduce XIAP degradation and decrease cisplatin-stimulated Caspase 3 activity [30,31]. Moreover, Akt phosphorylate Ataxin1 and modulate neurodegeneration 14-3-3 protein, thereby slowing its normal degradation [32]. As the ubiquitination of mannose receptor mediate the endosomal relocation of internalized antigens [4], the effect that Akt+ IKKα/β+ signalosome on the cross-presentation might attribute to phosphorylated Akt/IKK attenuated mannose receptor ubiquitination and antigen degradation, which occurs in Rab5+ endosome.

Akt, a pivotal signal transducer for growth and survival, can be activated at Thr-308 and Ser-473 regulatory sites [33,34]. Balasuriya et al. found that Thr-308 phosphorylation increase Akt1’s catalytic rate 1500-fold [35], confirming that Thr-308 phosphorylation, but not Ser-473, is required for Akt activation. Nevertheless, Mao et al. revealed that Ser473 phosphorylation regulate both ciliary synthesis/assembly and disassembly, whereas pAKT-Thr308 determine the ciliary length [36]. In the present study, in despite the fact that the loading of endotoxin-free antigen induce Akt phosphorylation at Thr-308, and promote the endosomal recruitment of phosphorylated Akt, the exact effect of Akt phosphorylation at Ser473 and its role in endotoxin-containing pathogen-derived cross-presentation in BMPC still need further investigations.

The Rel/NF-κB family of transcription factors sequester in the cytosol of un-stimulated cells via non-covalent interactions with IκB [37]. Upon stimulation, IκB protein is firstly phosphorylated by the IKK and then degradated by the proteasome [37]. Whereas IKK was phosphorylated by Akt and exerted its effect on NF-κB activation [38], IKK complex can be phosphorylated via TGFβ-activated kinase 1 (TAK1) by binding bacterial/viral recognized TLRs [39], indicating that both Akt phosphorylation and the binding of TLRs are important events for pathogen-induced NF-κB activation. Walker et al. described an optimized laboratory procedure to isolate individual organelles during different stages of endocytosis by performing subcellular fractionation [40]. In the present study, in despite the fact that endotoxin-containing pathogen induce Akt/IKK phosphorylation and promote the formation of Akt+ IKKα/β+ signalosome, the exact effect of endotoxin-containing pathogen on endosomal trafficking in BMPC still need subcellular fractionation for further investigation.

Apart from the mitogen-activated protein kinase (MAPK) as well as the PI3K-Akt-mTOR (the mammalian target of rapamycin)-p70 S6 pathways [41], TLRs engage a set of myeloid differentiation factor 88 (MyD88) adaptor family members, including MyD88, MyD88-adaptor-like/TIR-associated protein (TIRAP), toll-receptor-associated activator of interferon (TRIF), and toll-receptor-associated molecule (TRAM), to activate IKK [42]. Whereas TRIF recruits to early endosomes to initiate the production of type Ⅰ interferon [42], TLR4-MyD88 signaling mediates the endosomal relocation of Sec61 in the process of the cross-presentation [6,19]. To explore the roles of TRIF and MyD88 in the formation of Akt+ IKKα/β+ signalosome and the endosomal relocation of Sec61, we incubated TRIF or MyD88 deficient BMPC with endotoxin-containing pathogen and monitored the endosomal relocations of phosphorylated Akt, IKKα/β, and Sec61α with confocal immunofluorescence microscope. As shown in Figure S7, the treatment with endotoxin-containing pathogen obviously increased the co-localized spots of phosphorylated Akt, IKKα/β, and Sec61α with Rab5. Importantly, the deficiencies of TRIF and MyD88 obviously abolished the co-localized spots of phosphorylated Akt, IKKα/β, or Sec61α with Rab5 (Figure S7). All these findings indicate that both TRIF and MyD88 contribute to the formation of Akt+ IKKα/β+ signalosome on Rab5+ endosomes and the endosomal relocation of Sec61 in BMPC.

The bindings of TLRs with ligands lead to the activation of p38, stress-activated protein kinase/Jun-amino-terminal kinase (SAPK/JNK) and NF-κB pathways [39]. Whereas TLR4 localize in the plasma membrane and sense lipids and proteins, TLR3/TLR7-9 localize in the endosome and recognize nucleic acids signal [43,44]. As TLR4-MyD88-IL-1 receptor-associated kinase (IRAK4) signaling is necessary for the endosomal relocation of TAP [6,15], the decreased cross-presentation by the inhibition of Myd88 + IRAK4+ Myddosome indicates that Myd88 + IRAK4+ Myddosome is pivotal for the process of cross-presentation [43]. We incubated MyD88 and IRAK4-deficient and control BMPC with endotoxin-containing pathogen, and assessed the cross-presented OVA with immunofluorescence microscope and cytometric analyses, respectively. The deficiencies of MyD88 and IRAK4 decreased the co-localized spots of cross-presented OVA with Rab5 (Figure S8). Flow cytometric analyses revealed that the cross-presented OVA was obviously inhibited by MyD88 and IRAK4 deficiencies (Figure S8). In the present study, in despite the fact that endotoxin-containing pathogen promote the formation of Akt+ IKKα/β+ signalosome, whether MyD88-IRAK4 signaling contribute to the formation of this Akt+ IKKα/β+ signalosome still need further investigations.

In the endosome-to-cytosol pathway, the endosomal recruitment of TAP is essential for the transport of internalized antigens from the endosome into the cytosol [6,15]. With a crucial tool that trapped Sec61 in the ER and prevented its recruitment into endosome, efficient cross-presentation was found requiring TLR4 dependent the endosomal recruitment of Sec61 [19]. These data shed light on a long-lasting question regarding antigen cross-presentation and point out that p97 and Sec61 provide the driving force for the transport [4,5,18,19,45]. In the present study, in despite that Akt+ IKKα/β+ signalosome mediate the endosomal relocation of Sec61, the role of Akt+ IKKα/β+ signalosome in endotoxin-containing pathogen-derived the endosomal recruitments of TAP and p97 still need further explorations.

Taken together, our data provide a new molecular mechanism for BMPC cross-presentation, which was mediated by the combining action of the endosomal recruitment of Akt+ IKKα/β+ signalosome and the relocation of Sec61 toward endosome. These findings indicate that Akt+ IKKα/β+ Rab5+ signalosome contribute to the endosomal recruitment of Sec61 and the superior cross-presentation efficacy of BMPC.

## Supplementary Materials

The following are available online at https://www.mdpi.com/2076-393X/8/3/539/s1, Figure S1: The treatment with ovalbumin induces the phosphorylations of Akt and IKKα/β, Figure S2: The silencing of Akt, IKKα, and IKKβ decreases endotoxin-containing pathogen-induced phosphorylation of Akt and IKKα/β, Figure S3: The treatments with wortmannin and Bay 11-7082 inhibit endotoxin-containing pathogen-derived cross-presentation in bone marrow precursor cells, Figure S4: The treatments with wortmannin and Bay11-7082 abolish endotoxin-containing pathogen-derived the endosomal relocation of phosphorylated Akt and IKKα/β, Figure S5: The pretreatments with wortmannin and Bay 11-7082 decrease the interaction of Sec61β with Rab5, Figure S6: The pretreatments with wortmannin and Bay 11-7082 inhibit the relocation of Sec61β, Figure S7: TRIF and MyD88 contribute to Akt+ IKKα/β+ signalosome formation on Rab5+ endosomes and the relocation of Sec61α toward endosomes, Figure S8: MyD88-IRAK4 augments antigenic cross-presentation in bone marrow precursor cells.
