# Supplementary material for: Akt+ IKKα/β+ Rab5+ Signalosome Mediate the Endosomal Recruitment of Sec61 and Contribute to Cross-Presentation in Bone Marrow Precursor Cells
Source: Vaccines (Basel). 2020 Sep 17;8(3):539. doi: 10.3390/vaccines8030539 (PMC7563657; doi:10.3390/vaccines8030539)
Supplement: Supplementary file 1 [file vaccines-08-00539-s001.pdf]

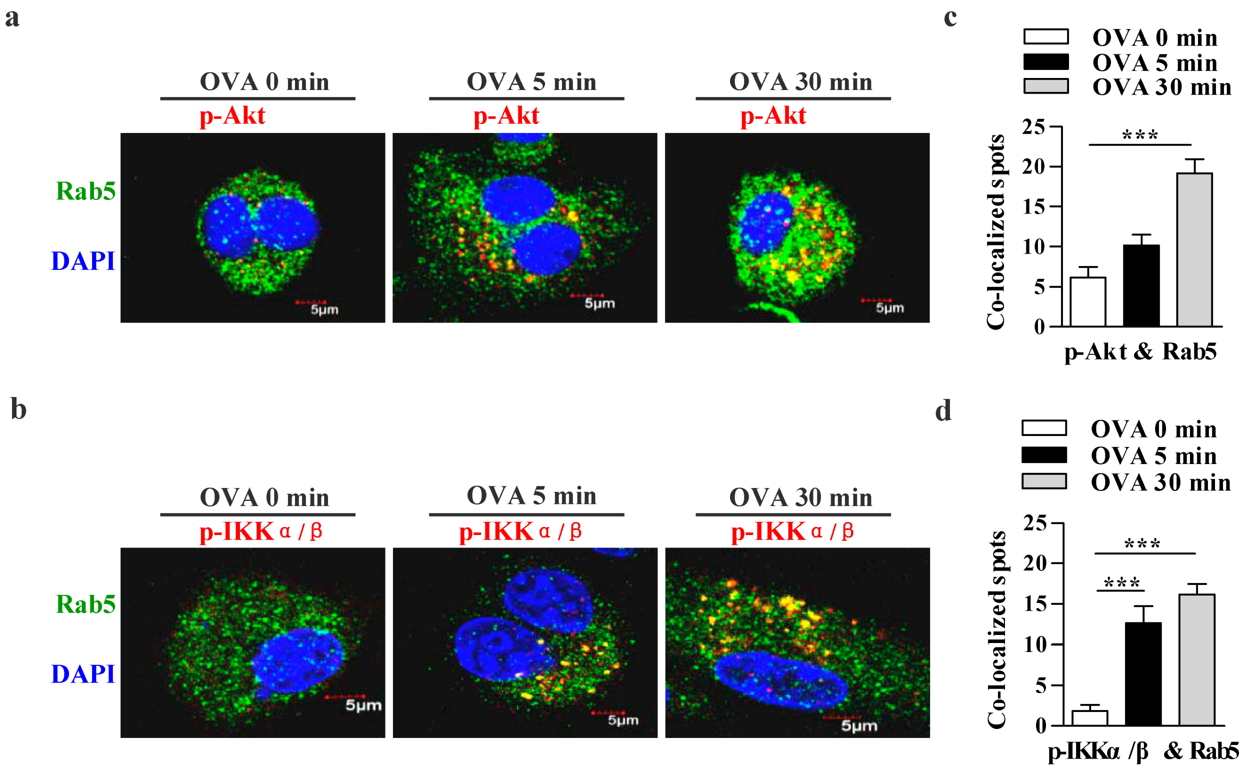

Figure S1. The treatment with ovalbumin induces the phosphorylations of Akt and IKKα/β. (**a–d**) Murine GM-CSF and IL-4 treated BMPC was incubated with ovalbumin (50 μg/mL) for indicated periods. The recruitment of phosphorylated Akt (**a**) and IKKα/β (**b**) toward endosomes was assessed by immuno-fluorescent microscope. phosphorylated Akt or IKKα/β was stained with phosphorylated antibodies (red); Rab5 (green); Nuclei were counterstained with DAPI (blue). The co-localized spots of Rab5 with phosphorylated Akt (**c**) and IKKα/β (**d**) were counted and analyzed. Original magnification, × 600. Data are presented as the mean±SEM, *** $p < 0.001$, one-way ANOVA with Newman-Keuls post test. One representative from 3 independent experiments is shown. Rab5: early endosome marker; OVA: ovalbumin.

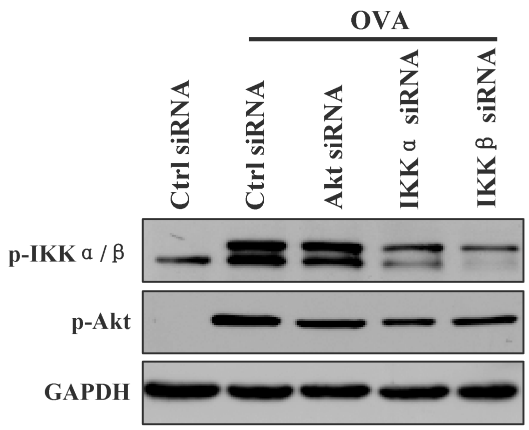

Figure S2. The silencing of Akt, IKKα and IKKβ decreases endotoxin-containing pathogen-induced phosphorylation of Akt and IKKα/β. Murine scramble, or Akt/IKKα/IKKβ deficient BMPC was incubated with endotoxin-containing pathogen ovalbumin (50 μg/mL). The whole cellular protein was extracted and the phosphorylation of Akt and IKKα/β was assessed by western blot. Control siRNA with ovalbumin was used as endotoxin-containing pathogen control. Control siRNA without ovalbumin was used as scramble control. GAPDH was used as protein loading control. One representative from 3 independent experiments is shown. OVA: endotoxin-containing pathogen ovalbumin.

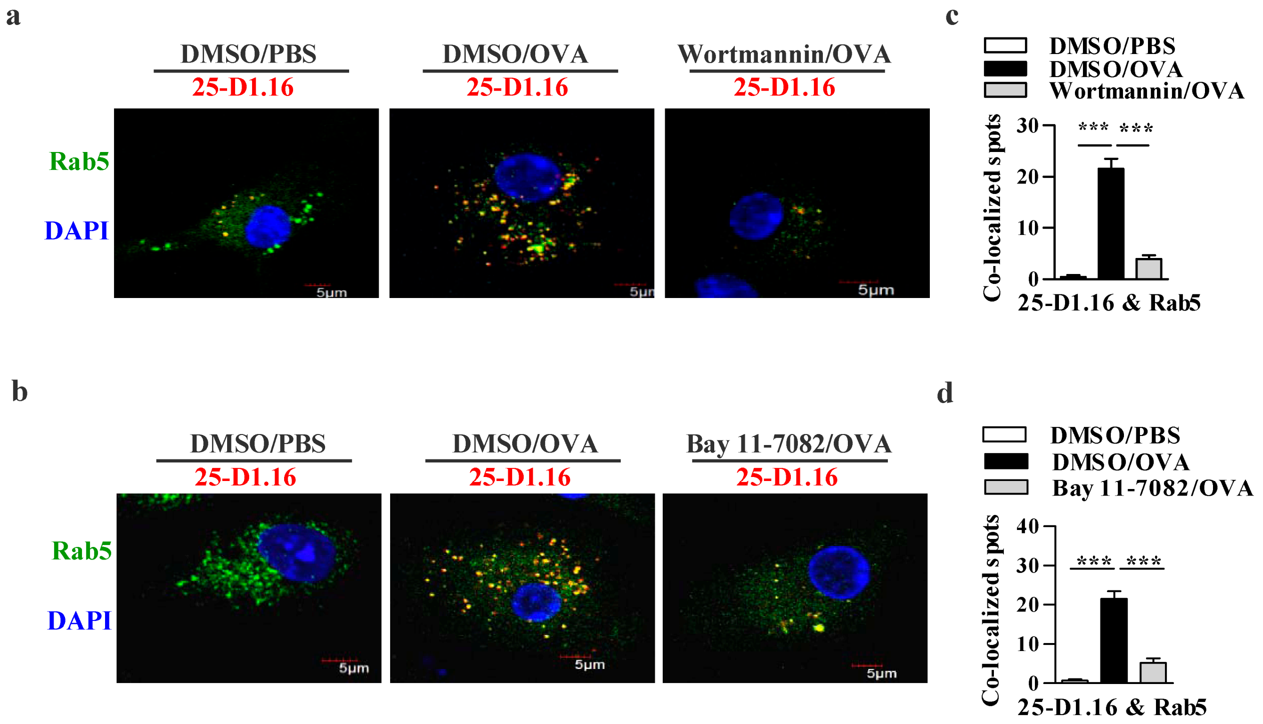

Figure S3. The treatments with wortmannin and Bay 11-7082 inhibit endotoxin-containing pathogen-derived cross-presentation in bone marrow precursor cells. Murine BMPC was pretreated with wortmannin (5 μmol/L) (**a**) or Bay11-7082 (5 μmol/L) (**b**) for 2 h prior to endotoxin-containing pathogen ovalbumin (50 μg/mL) incubation. The cross-presented OVA was observed with immuno-fluorescent microscope. Cross-presented OVA and Rab5 was stained with 25-D1.16 (red) or Rab5 antibody (green) respectively; nuclei were counter stained with DAPI (blue). The co-localized spots of 25-D1.16 with Rab5 were counted and analyzed (**c**,**d**). Data are presented as the mean±SEM, *** $p < 0.001$, one-way ANOVA with Newman-Keulspost test. One representative from 3 independent experiments is shown. Rab5: early endosome marker; OVA: endotoxin-containing pathogen ovalbumin.

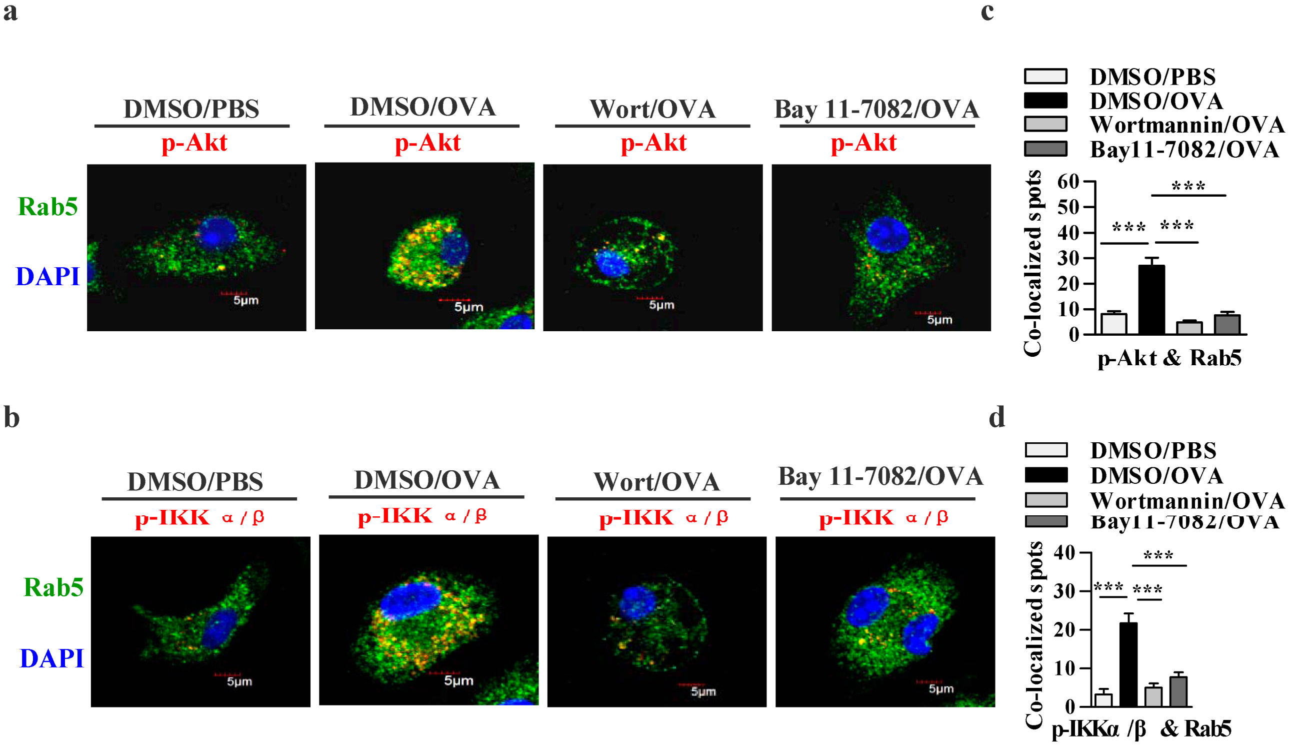

Figure S4. The treatments with wortmannin and Bay11-7082 abolish endotoxin-containing pathogen-derived the endosomal relocation of phosphorylated Akt and IKKα/β. (**a–d**) Murine BMPC was pretreated with wortmannin (5 μmol/L) (**a**) or Bay11-7082 (5 μmol/L) (**b**) prior to endotoxin-containing pathogen ovalbumin (50 μg/mL) incubation and the relocation of phosphorylated Akt (**a,c**) and phosphorylated IKKα/β (**b,d**) was assessed by immuno-fluorescent microscope with related antibody staining. phosphorylated Akt (**a**), phosphorylated IKKα/β (**b**) were stained red; Rab5 was stained green; nuclei were counter stained with DAPI (blue). The co-localized spots of Rab5 with phosphorylated Akt (**c**), phosphorylated IKKα/β (**d**) were counted and analyzed. Data are presented as the mean±SEM, *** $p < 0.001$, one-way ANOVA with Newman-Keulspost test. One representative from 3 independent experiments is shown. Rab5: early endosome marker; OVA: endotoxin-containing pathogen ovalbumin.

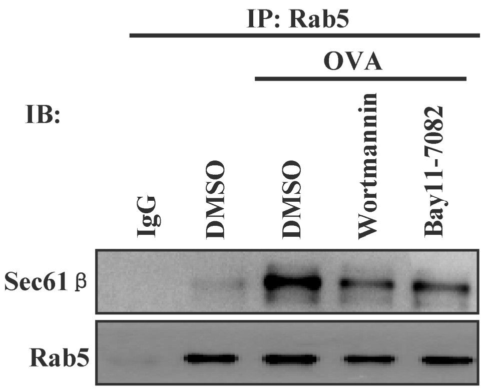

Figure S5. The pretreatments with wortmannin and Bay 11-7082 decrease the interaction of Sec61β with Rab5. Murine BMPC was pretreated with Bay11-7082/wortmannin (5 μmol/L) prior to endotoxin-containing pathogen ovalbumin (50 μg/mL) incubation. The interaction of Sec61β with Rab5 was investigated by Co-IP with Rab5 antibody. Isotype IgG was used as negative control. DMSO with ovalbumin was used as endotoxin-containing pathogen control. DMSO without ovalbumin was used as scramble control. Rab5: early endosome marker; OVA: endotoxin-containing pathogen ovalbumin.

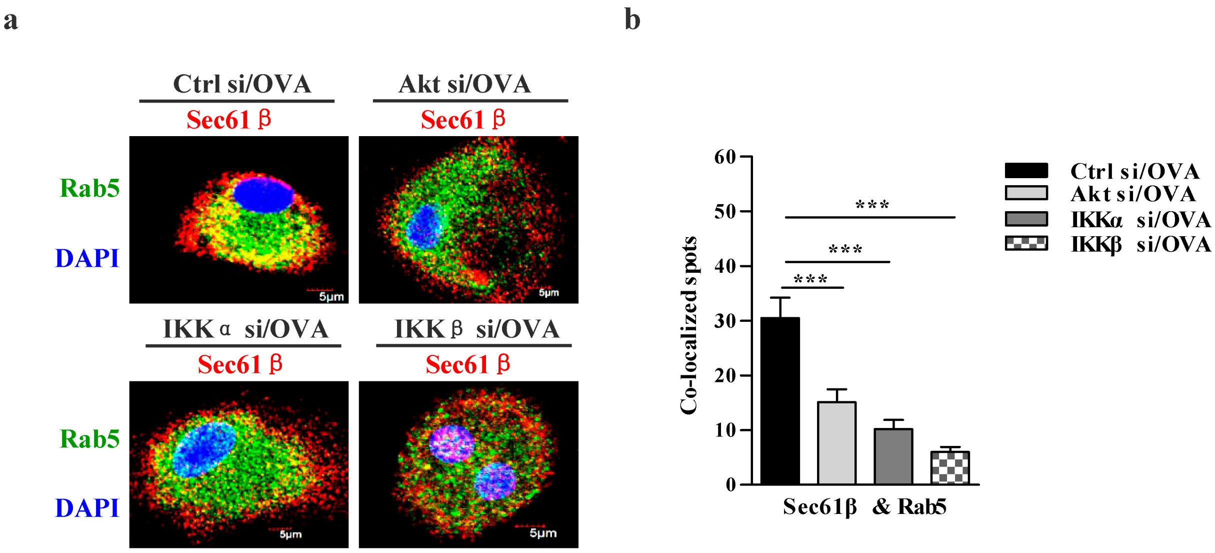

Figure S6. The pretreatments with wortmannin and Bay 11-7082 inhibit the relocation of Sec61β. Murine scramble, or or Akt/IKKα/IKKβ deficient BMPC was incubated with endotoxin-containing pathogen ovalbumin (50 μg/mL). The relocation of Sec61β with Rab5 (**a**) was assessed by confocal microscope with related antibody staining. Sec61β (**a**) was stained red; Rab5 was stained green; nuclei were counter stained with DAPI (blue). The co-localized spots of Rab5 with Sec61β (**b**) were

counted and analyzed. Data are presented as the mean±SEM, *** $p < 0.001$, one-way ANOVA with Newman-Keulspost test. One representative from 3 independent experiments is shown. Rab5: early endosome marker; OVA: endotoxin-containing pathogen ovalbumin.

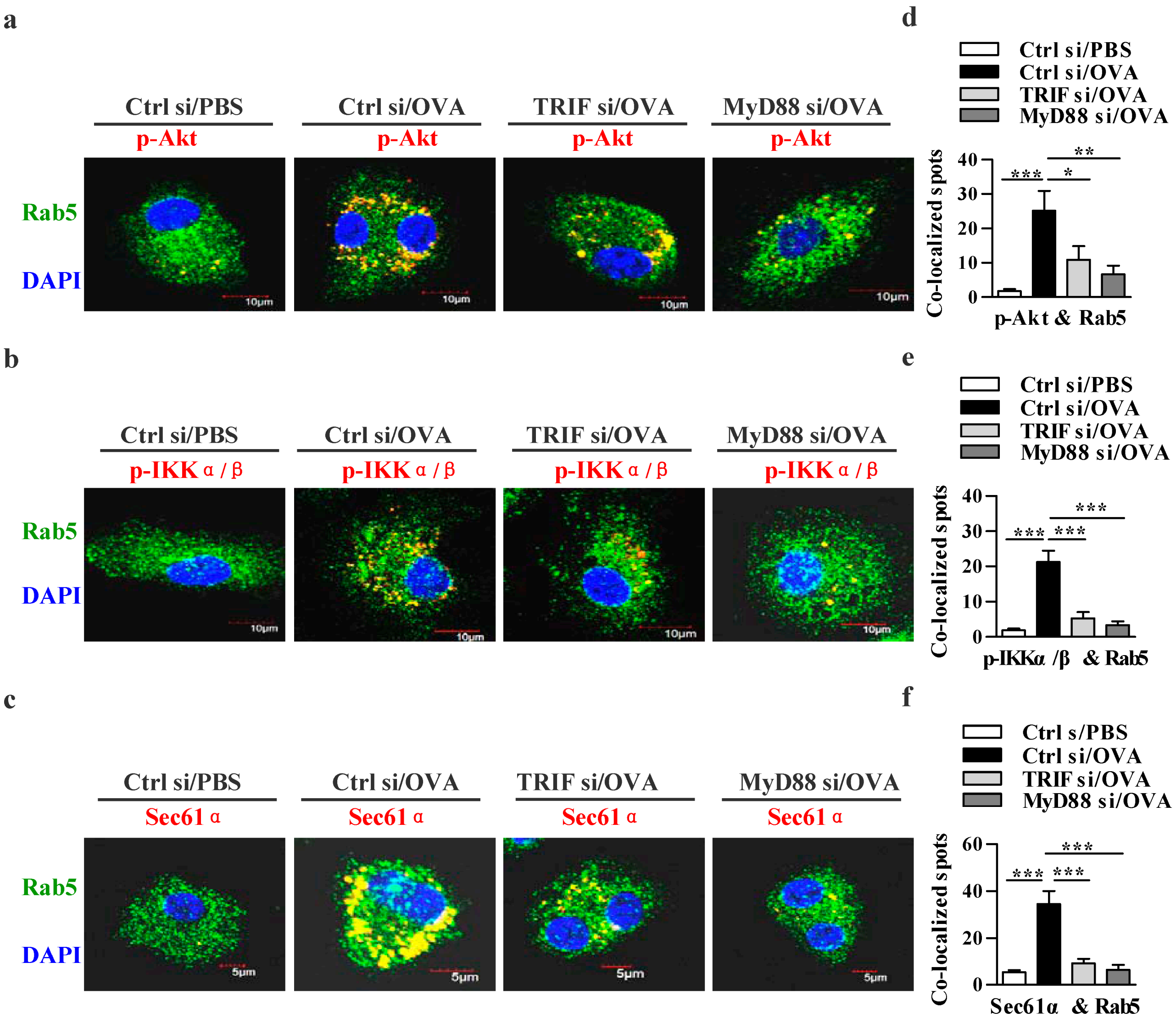

Figure S7. TRIF and MyD88 contribute to Akt+IKKα/β+ signalosome formation on Rab5+ endosomes and the relocation of Sec61α toward endosomes. TRIF or MyD88 deficient BMPC was incubated with ovalbumin (50 μg/mL) and the formation of Akt+IKKα/β+ signalosome (**a**,**b**) or the relocation of Sec61α toward endosomes (**c**) were assessed by immuno-fluorescent microscope. phosphorylated Akt (**a**), phosphorylated IKKα/β (**b**) and Sec61α (**c**) were stained red; Rab5 was stained green; nuclei were counterstained with DAPI (blue). The co-localized spots of Rab5 with phosphorylated Akt (**d**), phosphorylated IKKα/β (**e**) and Sec61α (**f**) were counted and analyzed. Original magnification, × 600. Data are presented as the mean±SEM, * $p < 0.05$, ** $p < 0.01$, *** $p < 0.001$, one-way ANOVA with Newman-Keulspost test. One representative from 3 independent experiments is shown. Rab5: early endosome marker; OVA: ovalbumin; si: siRNA.

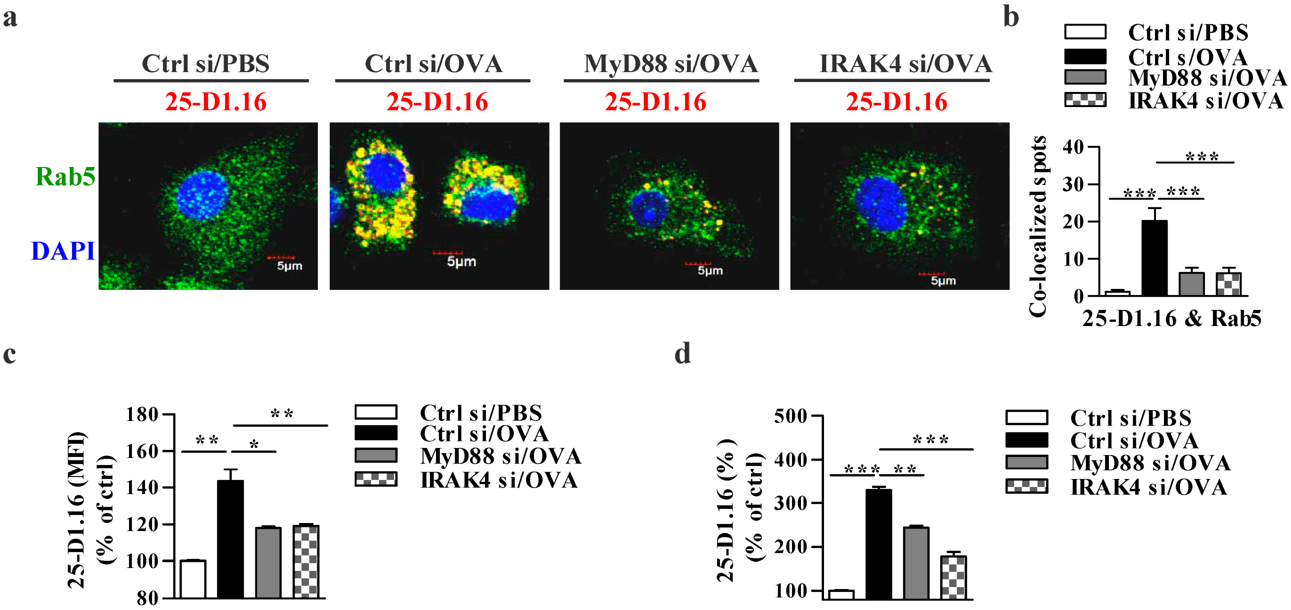

Figure S8. MyD88-IRAK4 augments antigenic cross-presentation in bone marrow precursor cells. (**a–d**) MyD88 or IRAK4 deficient BMPC was incubated with ovalbumin (50 μg/mL) and the effect of MyD88/IRAK4 deficiency on cross-presentation (**a–d**) was assessed by immuno-fluorescent microscope (**a**) and flow cytometric analyses (**c,d**), respectively. For immuno-fluorescent microscope, cross-presented OVA was stained with 25-D1.16 (red); Rab5 was stained green; nuclei were counter stained with DAPI (blue). The co-localized spots of Rab5 with 25D1.16 (**b**) was counted and analyzed. Original magnification, × 600. Data are presented as the mean±SEM, ** $p < 0.01$, *** $p < 0.001$, one-way ANOVA with Newman-Keulspost test. One representative from 3 independent experiments is shown. Rab5: early endosome marker; OVA: ovalbumin; si: siRNA.
